# Rapid On-Demand Point-of-Care Monitoring of Clozapine and Its Metabolite Norclozapine Using Miniature Mass Spectrometry

**DOI:** 10.3390/ph18101549

**Published:** 2025-10-14

**Authors:** Xiaosuo Wang, Wei Yi Lew, Yang Yang, Nan Zhang, Jiexun Bu, Zhentao Li, Michael Fitzpatrick, Paul Bonnitcha, David Sullivan, Wenpeng Zhang, Yu Zheng, John F. O’Sullivan

**Affiliations:** 1 Cardiometabolic-Medicine Laboratory, Charles Perkins Centre, The University of Sydney, Sydney, NSW 2006, Australia; weiyi.lew@sydney.edu.au; 2 School of Medical Sciences, Faculty of Medicine and Health, The University of Sydney, Sydney, NSW 2006, Australia; 3 Key Laboratory of TCM Clinical Pharmacy, Shenzhen Bao’an Authentic TCM Therapy Hospital, Shenzhen 518100, China; yangyanghb@outlook.com; 4 PURSPEC Technology (Beijing) Ltd., Beijing 100084, China; nan.zhang@purspec.cn (N.Z.); jiexun.bu@purspec.cn (J.B.); 5 Department of Pharmacy, The First Affiliated Hospital, Nanchang University, Nanchang 330006, China; ndyfy09785@ncu.edu.cn; 6 Chemical Pathology, NSW Health Pathology, Royal Prince Alfred Hospital, Camperdown, NSW 2050, Australia; michael.fitzpatrick1@health.nsw.gov.au (M.F.); paul.bonnitcha@health.nsw.gov.au (P.B.); david.sullivan@sydney.edu.au (D.S.); 7 NHMRC Clinical Trials Centre, Sydney Medical School, The University of Sydney, Sydney, NSW 2006, Australia; 8 State Key Laboratory of Precision Measurement Technology and Instruments, Department of Precision Instrument, Tsinghua University, Beijing 100084, China; zhangwp@tsinghua.edu.cn; 9 Mental Health Service, Croydon Health Centre, Sydney Local Health District, Croydon, NSW 2132, Australia; yu.zheng@health.nsw.gov.au; 10 Department of Cardiology, Royal Prince Alfred Hospital, Sydney, NSW 2050, Australia

**Keywords:** miniature mass spectrometry, approachable, clozapine/norclozapine, point-of-care testing, therapeutic drug monitoring, schizophrenia, real-time analysis

## Abstract

**Background/Objectives**: Clozapine remains the gold standard for treatment-resistant schizophrenia. However, its narrow therapeutic window and risk of severe side effects require close monitoring of both clozapine and its primary metabolite, norclozapine. Existing therapeutic drug monitoring (TDM) methods are limited by delays, high costs, and operational complexity. This study introduces three rapid point-of-care (POC) assays utilizing a miniature mass spectrometer (Mini-MS) to quantify clozapine and norclozapine in plasma, whole blood, and dried blood spots (DBSs), facilitating applications across diverse clinical settings. **Methods**: The analytical performance of the assay was evaluated for sensitivity, specificity, reproducibility, and correlation with reference methods. Clinical samples from two hospitals were analysed and validated against conventional liquid chromatography tandem mass spectrometry (LC-MS/MS) reference standards at New South Wales Health Pathology (NSWHP) and Tsinghua University laboratories. **Results**: The Mini-MS assay accurately quantified both analytes within therapeutic ranges across all matrices. Inter-assay coefficients of variation ranged from 7.9 to 14.1% for clozapine and from 1.6 to 14.6% for norclozapine. Accuracy fell between 85 and 117% in plasma and blood extracts. Strong linearity was demonstrated (R^2^ = 0.98–0.99) over the concentration range of 10–1000 ng/mL. Results from the Mini-MS analysis showed excellent correlations with LC-MS/MS results (r = 0.998). **Conclusions**: In this proof-of-concept study, the Mini-MS-based POC assays enable rapid, reliable quantification of clozapine and norclozapine, with performance comparable to conventional laboratory methods. This platform supports real-time TDM, facilitating timely dose adjustments, adherence monitoring, and ultimately improving patient outcomes.

## 1. Introduction

Clozapine, an antipsychotic medication, has been a cornerstone in the treatment of severe mental health disorders, particularly treatment-resistant schizophrenia (TRS), since its discovery in the 1950s and clinical introduction in the 1960s [1]. Schizophrenia is the most debilitating psychiatric disorder, affecting roughly 1 in 100 people globally, and manifests through a mix of delusions, hallucinations, thought disorders, and behavioural deficits [2]. Patients with schizophrenia face significant life-long challenges compounded by limited effective medication options.

Clozapine was initially identified as a breakthrough due to its unique efficacy in TRS but was withdrawn in 1975 due to safety concerns [3]. In 1989, however, the FDA reapproved clozapine for its unmatched effectiveness in managing TRS [4]. Notably, it remains the only FDA-approved medication for reducing suicidality in individuals with schizophrenia or schizoaffective disorder, offering substantial symptom relief for patients who do not respond to other therapies [5].

Despite its proven efficacy, clozapine therapy carries considerable risks. Common adverse effects include cardiometabolic disorders, severe neutropenia, and seizures [6,7,8,9]. Patients treated with clozapine face elevated risks of uncontrolled type 2 diabetes and pneumonia [6,10,11,12]. Furthermore, its complex metabolism, predominantly through CYP1A2 and CYP3A4, contributes to significant inter-individual variability in clozapine clearance rates [13]. Factors such as gender, ethnicity, smoking habits, and other pharmacological interactions affect clozapine’s metabolism, with further contributions from physiological conditions such as inflammation or obesity [14]. For example, smoking cessation requires a 30% dose reduction to maintain therapeutic levels of the drug [15,16].

Clozapine’s narrow therapeutic window (350−600 ng/mL for steady-state levels) requires close and regular monitoring to manage the associated risk of neutropenia and cardiovascular complications [17]. Norclozapine, the primary metabolite of clozapine, has a half-life approximately 1.5 to 2 times longer than clozapine’s and is less sensitive to transient fluctuations [18]. Hence, monitoring both analytes captures short-term changes and long-term trends, supporting adherence assessment, dose optimization, and the reduction of dose-related adverse drug reactions (ADRs) [19,20]. Non-adherence, in turn, may compromise treatment efficacy, increase sensitivity to clozapine’s toxicities, and elevated risk of self-harm [20,21]. To mitigate these risks, clozapine initiation requires mandatory weekly dose adjustments and blood tests for the first 18 weeks, followed by monthly monitoring at a dedicated clozapine clinic [22,23]. Given the interplay of genetic variability and environmental influences, predicting clozapine metabolism remains highly challenging. Thus, real-time or near real-time access to clozapine levels is essential for timely and informed clinical decision-making.

However, current therapeutic drug monitoring (TDM) protocols dependent on conventional liquid chromatography tandem mass spectrometry (LC-MS/MS) present logistical, financial, and time-related challenges [24]. In Australia, result turnaround can take several days, reducing clinical utility, delaying treatment adjustments, and increasing uncertainty due to sample degradation [14]. These monitoring demands raise healthcare costs and create access barriers, particularly in resource-limited settings. In urgent cases, rapid titration of clozapine may be necessary to stabilize patients quickly, but this increases the risk of seizures, myocarditis, and hypotension which further complicates management due to the lack of real-time monitoring [25]. In cases of suspected noncompliance, the consequences can be life-threatening, ranging from toxicity due to loss of tolerance when treatment is abruptly resumed to the return of suicidality as clozapine’s protective effects diminish. These time-sensitive scenarios highlight the urgent need to reduce assay turnaround times to support the safe and effective use of clozapine [15].

In recent years, miniature mass spectrometry (Mini-MS) systems have garnered attention for point-of-care (POC) testing development since the invention of the discontinuous atmospheric pressure interface (DAPI) [26]. DAPI technology implemented with a pulsed nano electrospray ionization (nESI) source improves sample usage efficiency and enhances ion transfer to an ion trap mass spectrometer (MS) [27]. With advances in Mini-MS, applications such as TDM, biomarker detection, and pharmacokinetic studies have been explored, including large-scale clinical trials [28,29].

In this study, we present near real-time POC assays for the rapid quantification of clozapine and norclozapine in plasma, fresh whole blood, and dried blood spots (DBSs) using a portable Mini-MS system. Plasma-based assays are generally the most reliable due to the simpler matrix and close comparability to conventional LC-MS/MS methods. However, the requirement for centrifugation limits their use to clinical or laboratory settings equipped with a centrifuge. Whole blood, while time-sensitive and requiring immediate processing, is well suited for on-site testing. DBS samples, by contrast, are stable, easy to store and transport, and suitable for patient self-collection with subsequent shipment to centralized laboratories or clinics. The workflow and results were compared with an existing protocol using a conventional LC-MS/MS system. These innovations could dramatically enhance the accessibility and safety of clozapine therapy by enabling real-time monitoring of clozapine and norclozapine levels, reducing healthcare costs and noncompliance burden, and allowing for more informed dose adjustments in acute clinical settings.

## 2. Results

### 2.1. Development and Performance of Clozapine and Norclozapine Detection Using Mini-MS

**Figure 1:**
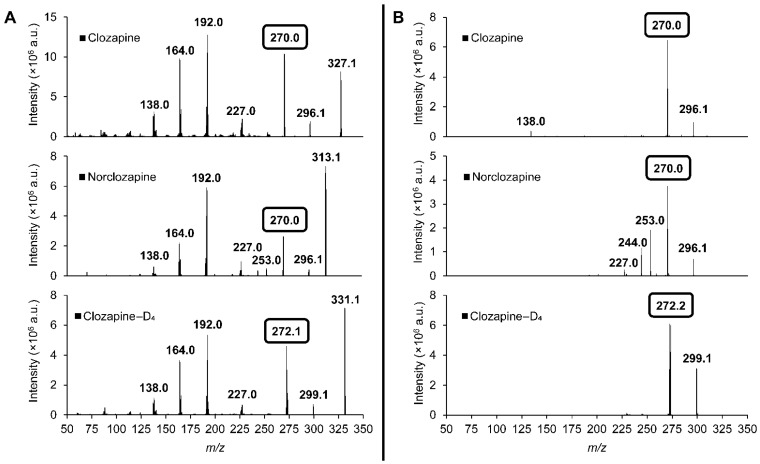
MS/MS spectra of clozapine and norclozapine obtained through two different methods: (**A**) conventional LC-MS/MS using a Sciex Triple Quad 7500 via syringe flow injection at 5 uL/min, and (**B**) Mini-MS system using a nano-electrospray ionisation cartridge with injection size set at 40. Product ions of clozapine (m/z 327 → 270), norclozapine (m/z 313 → 270) and clozapine-D_4_ (m/z 331 → 272) were monitored for quantification purposes.

Standards of clozapine, norclozapine, and clozapine-D_4_ were first scanned for its precursor and product ions using a nESI cartridge in the Mini-MS system. The detected ions were consistent with those obtained from Q3 MS1 and product ion scans performed on a conventional LC-MS/MS system using a syringe infusion of the analytes (Figure 1A,B). The prominent product ions of m/z 327 → 270, 313 → 270 and 331 → 272 were used as quantifiers for clozapine, norclozapine and clozapine-D_4_, respectively, on both instruments.

**Figure 2:**
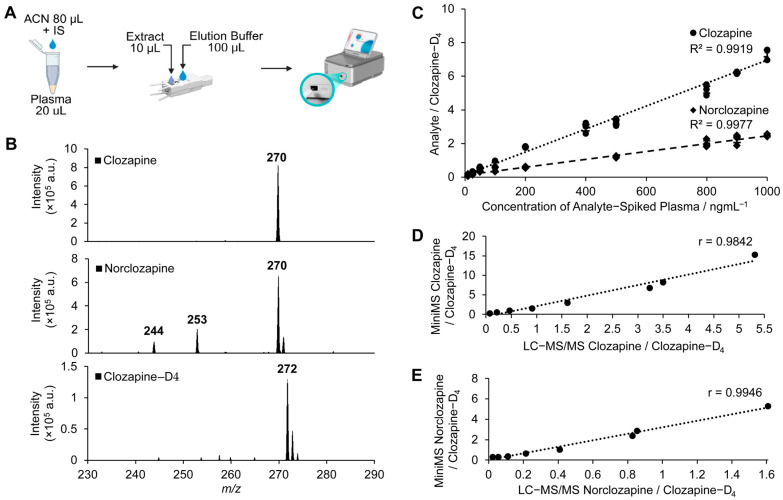
Rapid analysis of clozapine and norclozapine in human plasma using a Mini-MS system. (**A**) Workflow diagram illustrating the one-step protein precipitation of plasma prior to application onto and elution through the paper capillary spray (PCS) cartridge for Mini-MS analysis. (**B**) MS/MS spectra of clozapine, norclozapine, and clozapine-D_4_ (spiked at 100 ng/mL) in human plasma, detected using the Mini-MS system. (**C**) Calibration curves for clozapine and norclozapine in human plasma. Serial concentrations were spiked into blank human plasma, with a constant amount of clozapine-D_4_ as an internal standard (IS). (**D**,**E**) Correlation plots comparing calibration curves for (**D**) clozapine and (**E**) norclozapine between the Mini-MS system and the Sciex Triple Quad 7500.

Next, plasma samples from healthy volunteers were spiked with internal standard (IS) and varying concentrations of clozapine and norclozapine. Following a one-step protein precipitation process, the extract was analysed on the Mini-MS (Figure 2A). The characteristic product ion m/z 270 of clozapine was clearly observed. For norclozapine, two other prominent ions m/z 253 and m/z 244 were also detected along with m/z 270 (Figure 2B). Linear regression analysis of the Mini-MS demonstrated a strong correlation between peak intensity ratios of clozapine and norclozapine relative to the IS and the varying concentrations of clozapine and norclozapine ranging from 10 to 1000 ng/mL (R^2^ > 0.99, Figure 2C). The same plasma extracts analysed in parallel on conventional LC-MS/MS demonstrated a strong correlation with data generated from the Mini-MS, showing a correlation coefficient (r) of 0.9842 and 0.9946 for clozapine and norclozapine, respectively (Figure 2D,E).

**Figure 3:**
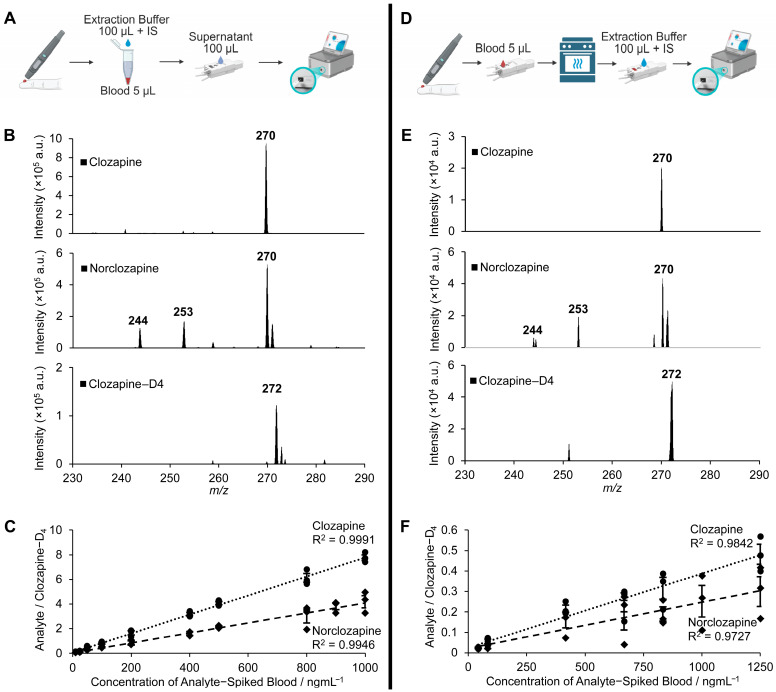
Rapid analysis of clozapine and norclozapine in human blood extracts and dried blood spots (DBSs) using PCS cartridges and a Mini-MS system. (**A**) Workflow diagram for the fast analysis of clozapine and norclozapine in one-step blood extracts. (**B**) MS/MS spectra of clozapine, norclozapine, and clozapine-D_4_ in blood extract. (**C**) Calibration curves for clozapine and norclozapine in blood extracts using clozapine-D_4_ as an internal standard (IS). (**D**) Workflow diagram for the rapid analysis of clozapine and norclozapine in DBS. (**E**) MS/MS spectra of clozapine and norclozapine-D_4_ in DBS samples. (**F**) Calibration curves for clozapine and norclozapine in DBS samples on paper cartridges using clozapine-D_4_ as the IS.

To further evaluate the Mini-MS system, fresh blood samples (5 µL) from healthy volunteers were spiked with IS and varying concentrations of clozapine and norclozapine and then extracted with buffer before analysis on the Mini-MS (Figure 3A). The MS/MS spectra of clozapine and norclozapine were well detected, though some chemical noise was observed (Figure 3B). Linear regression curves were generated, with strong R^2^ values for both clozapine (R^2^ = 0.9991) and norclozapine (R^2^ = 0.9946) across 10–1000 ng/mL (Figure 3C). Compared to the plasma calibration curves, however, peak intensity ratios were decreased consistently by 26–29% in the whole blood extracts due to the more complex matrix of blood.

To assess the Mini-MS’s capability for POC testing, we also analysed DBS samples. Blood (5 µL) spiked with various concentrations of clozapine and norclozapine was spotted directly onto paper capillary spray (PCS) cartridges and eluted with IS-spiked extraction buffer for Mini-MS analysis (Figure 3D). MS/MS spectra of all compounds were consistent with previous matrices, though with reduced intensity and accompanied by small chemical noise (Figure 3E). Strong linearity was established for clozapine (R^2^ = 0.9842) and norclozapine (R^2^ = 0.9727) (Figure 3F).

The performance of this POC assay was systematically evaluated for reproducibility and accuracy, as summarized in Table 1. Intra-assay coefficients of variation (CVs) were below 10% across most concentrations in both plasma and whole-blood matrices. Inter-assay measurements for clozapine and norclozapine in both plasma and blood extracts were below 15% over 12 months. Notably, at 166.7 ng/mL in DBS, the inter-assay CVs were 9.6% and 17.8% for clozapine and norclozapine, respectively, based on four experiments conducted over four weeks (Table 1).

The limit of quantification (LOQ), as determined from the calibration curves, was 10 ng/mL for clozapine and norclozapine in plasma and whole blood extracts, and 41.7 ng/mL in DBS. The corresponding limits of detection (LODs) were 0.5 ng/mL in plasma and whole blood extracts and 2.5 ng/mL in DBS. Representative MS/MS spectra are provided in Figure S2.

The efficiency of the one-step solvent extraction was evaluated by comparing neat analyte concentrations to those spiked into plasma or blood samples. Average recoveries for both matrices ranged from 60% to 81%. The assay exhibited high precision and accuracy across three different concentrations, as shown in Table 1. Stability testing over a 24 h period in both plasma and blood samples showed a minimal variation of 4%.

### 2.2. Clinical Applications in Confirmed Cases at Two Hospital Sites

Blood samples from 25 patients undergoing clozapine therapy were first analysed at Tsinghua University using a conventional LC-MS/MS system. In parallel, the same samples were analysed using the Mini-MS platform, with final concentrations presented in Table S1. With clozapine, notably, sample 8 was the only case of non-detection in both the LC-MS/MS and the Mini-MS, potentially indicating non-adherence. Deviations between the two instruments ranged from 0.05 to 12.53%. With norclozapine, samples 4, 8, 9, and 20 were undetectable by both instruments, and deviations ranged from 0.22 to 13.42%.

**Figure 4:**
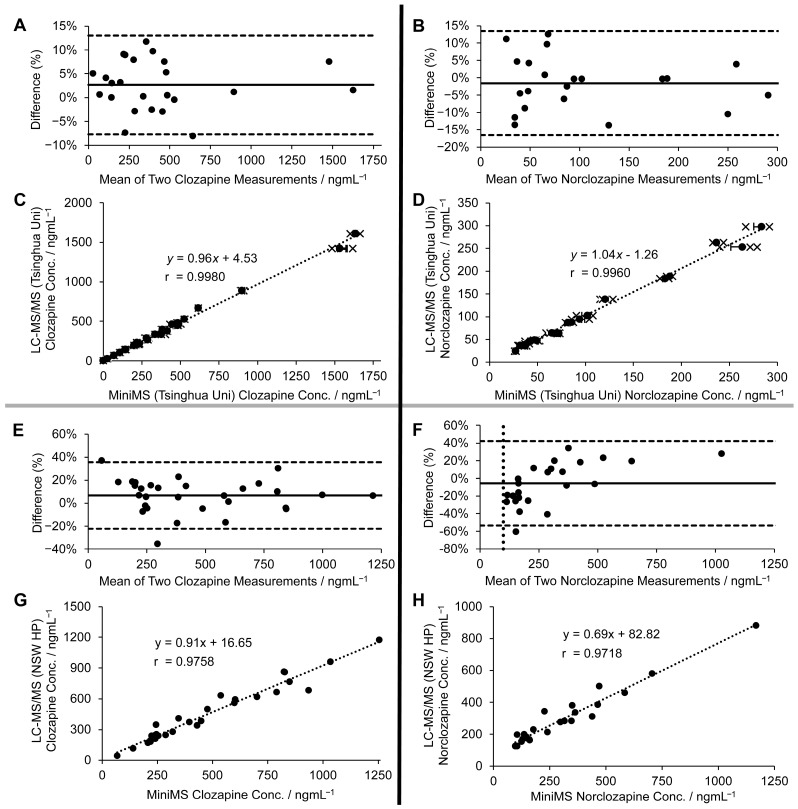
Comparison of levels of clozapine and norclozapine in clinical samples analysed by the conventional LC-MS/MS and the Mini-MS system. (**A**,**B**) Bland–Altman plots comparing (**A**) clozapine and (**B**) norclozapine measurements made between the two methods at Tsinghua University. The dotted line represents the mean difference between two measurements. (**C**,**D**) Scatterplot showing correlation of (**C**) clozapine and (**D**) norclozapine measurements made between the LC-MS/MS and the Mini-MS system at the Tsinghua University laboratory. (**E,F**) Bland–Altman plots comparing (**E**) clozapine and (**F**) norclozapine measurements made between the conventional LC-MS/MS system at the New South Wales Health Pathology (NSWHP) Laboratory and the Mini-MS system at the University of Sydney Laboratory. (**G**,**H**) Scatterplot showing correlation of (**G**) clozapine and (**H**) norclozapine measurements made between the LC-MS/MS at NSWHP and the Mini-MS system at the University of Sydney laboratory.

To assess the agreement between Mini-MS and LC-MS/MS performed at Tsinghua University laboratory, Bland–Altman plots were generated for clozapine and norclozapine (Figure 4A,B), demonstrating excellent concordance. The mean difference ranged from −8% to 13% for clozapine (bias: 2.7%, width: 10.4%, 95% CI) and from −17% to 13% for norclozapine (bias: −1.6%, width: 15.0%, 95% CI). Correlation analysis further supported this agreement, with r = 0.998 for clozapine and r = 0.996 for norclozapine (Figure 4C,D).

Following the single-centre evaluation, Mini-MS performance was further assessed in a two-centre setting to highlight operator differences, protocol variations, and sample ageing. Serum samples from 30 patients were first analysed at New South Wales Health Pathology (NSWHP) laboratory using the standard conventional LC-MS/MS protocol, after ethics clearance, then re-analysed three weeks later at the University of Sydney laboratory using Mini-MS. Bland–Altman analysis showed acceptable but weaker agreement between the two analysis, with limits of agreement of −22% to 36% for clozapine (bias: 6.8%, width: 29.0%, 95% CI) and −53% to 42% for norclozapine (bias: −5.5%, width: 47.8%, 95% CI) (Figure 4E,F). Correlation coefficients between the two centres remained high, though reduced compared with the single-centre study (r = 0.9758 for clozapine and r = 0.9718 for norclozapine; Figure 4G,H). Full measurement data are presented in Table S2.

## 3. Discussion

In this report, we present a rapid, reliable POC detection method using a Mini-MS system that enables accurate quantification of clozapine and norclozapine across plasma, blood, and DBS samples. Method validation was performed against conventional LC-MS/MS by confirming precursor and product ion identities and by evaluating the correlation coefficients from regression analyses and clinical sample data.

Clozapine, regarded as the most effective treatment for patients with schizophrenia, is often underutilized in clinical practice [30]. Intensive TDM of clozapine and its metabolite norclozapine is essential to guide individualized dosing, optimize therapeutic benefit, and reduce the risk of dose-dependent adverse effects [17]. However, timely treatment initiation and dose adjustment are frequently hindered by delays in drug analysis, with one of the most critical barriers being the lack of reliable real-time measurement of clozapine concentrations [31,32]. The rapid detection capabilities of Mini-MS therefore represent a promising solution for both emergency and routine clinical settings.

In this proof-of-concept study, the developed Mini-MS POC assay demonstrated reliable sensitivity and precision, with intra-assay CVs consistently < 10% across most concentrations and inter-assay variability limited to the lower quantification range well below the therapeutic window (350–600 ng/mL) [33,34]. Robustness was further confirmed by reproducible CVs collected over twelve months within the assay’s working range.

Average recovery across plasma, whole blood, and DBSs was modest, reflecting the simplicity of the one-step extraction. The potential matrix effects were effectively controlled using calibration curves in blank matrices covering the clinically relevant range, together with a low concentration of stable isotope-labelled IS to compensate and normalise variability [26,35]. The 13 ms ion pulse width of the DAPI with nESI source [36] further limited ion sampling, reducing potential matrix effects and co-elution issues compared with traditional LC interfaces. Sample loading can be increased to enhance sensitivity if required.

Clinical feasibility was evaluated at two independent locations. At Tsinghua University, Mini-MS measurements from patient blood samples demonstrated a strong and statistically significant correlation with that in conventional LC–MS/MS. Further Bland–Altman analysis confirmed excellent agreement across a 25-patient cohort between two analyses. After adjusting for minor bias, the level of agreement was within clinical threshold, with ±10.4% for clozapine and ±15.0% for norclozapine, supporting the potential utility of Mini-MS for TDM. Notably, sample 8 was undetectable by both platforms, suggesting potential non-adherence to clozapine therapy.

Non-adherence poses serious risks for patients with TRS [37]. Equally importantly, once treatment is initiated, careful dose titration and monitoring for adverse effects, particularly cardiovascular complications, are essential to maintain both safety and therapeutic efficacy [18]. Both challenges highlight the need for frequent and accessible drug monitoring. In this context, the Mini-MS assays offer a practical solution, enabling rapid detection of clozapine and norclozapine to assess adherence, support individualized dose adjustments, and guide patient-tailored interventions based on specific risk factors.

Next, in the Sydney cohort, the importance of method harmonization and standardization was further demonstrated. The wider limits of agreement observed in the Sydney cohort largely reflect methodological heterogeneity (different chemical standards, extraction protocols, instruments, and a three-week delay between analyses due to ethical and logistical constrains), rather than the intrinsic performance of Mini-MS. Importantly, correlation between platforms remained high across sites, underscoring the robustness of Mini-MS. To advance clinical implementation for real-time TDM, non-adherence assessment, and pharmacokinetic studies in diverse practice environments, continued harmonization of experimental methods is required.

Beyond analytical performance, the Mini-MS assay offers clear clinical and economic advantages. By reducing reliance on centralized laboratory workflows, the assay has the potential to lower processing costs, shorten turnaround times, and reduce hospital stays. Faster reporting of clozapine levels would enable more responsive and individualized dose adjustments, potentially lowering the incidence of dose-related ADRs while improving symptom control and reducing the psychosocial burden of uncontrolled psychosis [25]. Moreover, the portability and speed of Mini-MS testing may expand access to clozapine therapy in resource-limited settings, where real-time monitoring could significantly improve treatment outcomes and patient safety.

This study has several limitations. The relatively small sample sizes (*N* = 25 and 30) and ethical restrictions limited access to diverse clinical samples, including haemolysed and lipemic plasma, preventing full assessment of matrix effects. Parallel analysis of identical serum extracts by both Mini-MS and conventional LC-MS/MS from the Sydney cohort was not feasible, limiting direct comparability, highlighting the need for method harmonization in future clinical applications. DBS-based assays were not fully validated with clinical samples, and further optimization is needed to streamline sample preparation for routine clinical use. Future studies with larger, more diverse cohorts, standardized protocols, and harmonized methods are required to confirm assay accuracy, robustness, and versatility. Additionally, cost–benefit and workflow integration analyses in clinical settings are necessary to support implementation, which were beyond the scope of this study.

## 4. Materials and Methods

### 4.1. Chemicals and Reagents

Analytical-grade chemical standards of clozapine (≥98%), internal standard (IS) clozapine-D_4_ (≥99%), and norclozapine (≥98%) were obtained from Merck (Darmstadt, Germany). LC-MS-grade solvents, including water, acetonitrile, isopropanol, methanol, ammonium formate, and formic acid were purchased from Thermo Fisher Scientific (Waltham, MA, USA). Paper capillary spray (PCS) calibration and development cartridges with fused silica capillaries, and nESI cartridges were sourced from PURSPEC Technology (Beijing, China). The detailed methods are presented in the Supplemental Materials.

### 4.2. Blood and Plasma Collection

Human blood and plasma samples were collected from healthy laboratory staff, with approval from the Human Research Ethics Committee in Sydney Local Health District. Patient blood samples (*N* = 25) were obtained at the First Affiliated Hospital of Nanchang University. The procedures were reviewed and approved by the Ethics Review Board of Tsinghua University, and informed consent forms were obtained from all participants. All samples were stored and analysed at Tsinghua University. Additional patient serum samples (*N* = 30) were obtained from the NSWHP Chemical Pathology Laboratory with ethics approval by the NSWHP Research Governance Office.

### 4.3. Preparation of Internal Standards, Calibration Curves, and Quality Control Samples

Clozapine-D_4_ (IS) was added (200 ng/mL) in all samples. Clozapine and norclozapine standards were prepared in methanol and spiked into biological matrices at various concentrations for calibration curves from 10 to 1000 ng/mL. Quality control (QC) samples were prepared at 100, 500, and 1000 ng/mL. For DBS, concentrations ranged from 41.7 to 1250 ng/mL, with QC levels at 166.7, 416.7, and 1250 ng/mL. A buffer of ammonium formate (5 mM) and formic acid (0.1%) was prepared in 98% acetonitrile/water for extraction and elution.

### 4.4. Preparations for Plasma, Whole Blood, and Dried Blood Spots

For plasma/serum samples, IS and analytes were spiked into 20 µL of plasma/serum. After adding acetonitrile to make the final volume to 100 µL, samples were vortexed and centrifuged. The supernatant was loaded and eluted with extraction buffer and analysed by both the Mini-MS and the LC-MS/MS.

For whole blood samples, 5 µL of blood was spiked with IS and analytes then made up to 105 µL with extraction buffer. After vortexing and brief centrifugation, the supernatant was loaded for the Mini MS analysis.

For DBS samples, blood (5 µL) spiked with varying concentrations of analyte was applied to a PCS cartridge, dried (37 °C, 1 min) and eluted with 100 µL of the extraction buffer containing IS for Mini-MS analysis.

### 4.5. Miniature Mass Spectrometry and Workflow

The Mini-MS system (Cell, PURSPEC Technology, Beijing, China) consists of a cartridge inlet, DAPI, linear ion trap, and detector [38]. It supports precursor and product ion scans in positive and negative modes (m/z 50–2000) [39]. Standard solutions (1 µM) were analysed to identify key transitions for clozapine (m/z 327 → 270), norclozapine (m/z 313 → 270), and clozapine-D_4_ (m/z 331 → 272).

For plasma, 10 µL of extract was loaded into PCS cartridges, followed by 100 µL of extraction buffer. Whole blood extracts were directly loaded (100 µL), and DBS (5 µL) samples were eluted with extraction buffer (100 µL) in cartridge before analysis. Ionisation was performed at 3.8–4.5 kV spray voltage. Collision energy was optimised, with scans conducted for MS1_ISO, MS2, and MS2_ISO modes. The total hands-on time for the analysis was approximately 3–4 min for all three assays; see Figure S1 for a breakdown of the workflow and detailed time estimates.

### 4.6. Liquid Chromatography Tandem Mass Spectrometry (LC-MS/MS)

A triple quadrupole 7500 mass spectrometer (AB Sciex, Foster City, CA, USA) coupled with a Nexera LC-30AD UHPLC (Shimadzu Corporation, Kyoto, Japan) system was used in positive electrospray ionization mode. Separation was achieved on an Agilent HPH C18 column using a gradient of water/acetonitrile with ammonium acetate. MRM transitions were clozapine (m/z 326.8 → 270, 192), norclozapine (m/z 313 → 270, 192), and clozapine-D_4_ (m/z 331 → 272). MS parameters included 3000 V spray voltage, 100 °C source temperature, and optimised gas settings, as detailed in the Supplemental Materials.

### 4.7. Performance Evaluation of Mini-MS

The specificity for detecting both precursor and product ions of each analyte using the Mini-MS was validated against the Sciex 7500 LC-MS/MS (AB Sciex, Foster City, CA, USA). Linearity for plasma, whole blood extracts and DBS samples was evaluated across a range of concentrations using the previously described preparation methods. The limit of quantification (LOQ) was defined by a coefficient of variation (CV) < 20% and accuracy within 80–120%. The limit of detection (LOD) was defined where S/N ≥ 3. [33,40] Inter- and intra-assay reproducibility were tested in triplicate across four days at low, medium, and high QC concentrations.

The extraction efficiency was calculated by comparing analyte responses in spiked matrices versus that in solvent. Accuracy tests were assessed as follows:

(1) $$\mathrm{Accuracy}\;\%\;=\frac{\mathrm{measured}\;\mathrm{concentration}}{\mathrm{nominal}\;\mathrm{concentration}}\times 100$$

### 4.8. Clozapine-Treated Patient Samples from Two Hospital Sites

Blood samples from patients (*N* = 25) on clozapine therapy were analysed using both Mini-MS and conventional LC-MS/MS platforms at Tsinghua University. For the Mini-MS, blood (100 µL) was extracted with 400 µL of acetonitrile containing IS. After brief vortexing, the mixture (100 µL) was loaded into the PCS cartridge for Mini-MS analysis. In parallel, the samples were centrifuged (12,000 rpm, 4 °C, 5 min). The resulting supernatant (200 µL) was filtered through a 0.22 µm PTFE filter for quantitative LC-MS/MS analysis in positive MRM mode using a Shimadzu 8040 LCMS system (Kyoto, Japan).

Patient serum samples (*N* = 30, 50 µL each) were obtained from the NSWHP Chemical Pathology Laboratory, Sydney, and initially analysed at the same laboratory using a Shimadzu 8050 LCMS system (Kyoto, Japan) operated in positive electrospray ionization mode. Each sample was extracted with 400 µL of acetonitrile containing the IS, subjected to phospholipid removal, centrifuged, and subsequently diluted prior to MRM analysis. Full methodological details are in the Supplemental Materials.

The same serum samples were then transferred to the University of Sydney laboratory and, following ethics clearance, re-analysed three weeks later using the Mini-MS platform. For Mini-MS analysis, 20 µL of serum was extracted with 80 µL of IS-spiked extraction buffer. After brief centrifugation, 10 µL of the resulting supernatant was used for measurement.

### 4.9. Data Analysis

Data retrieved from the Mini-MS was analysed using PMS Client Pro software (version 3.6.2.2, PURSPEC Technology, Beijing, China) and exported to Excel for further analysis. Data from the LC-MS/MS was analysed using Sciex OS (version 3.1, AB Sciex). Analyte signals were normalised to IS signals using peak height and peak area in Mini-MS and LC-MS/MS data, respectively, before calibration curves were constructed and quantifications performed.

## 5. Conclusions

In conclusion, while clozapine remains the gold standard for TRS, its use is hindered by significant monitoring challenges. The Mini-MS system could overcome these barriers by enabling accessible POC monitoring in various matrices, improving therapy management, reducing costs, and enhancing outcomes for patients reliant on this life-saving drug. We developed and validated a Mini-MS based platform for rapid, near POC monitoring of clozapine and its primary metabolite, norclozapine, across plasma, whole blood, and DBS matrices. The assay demonstrated robust linearity within the therapeutic range, reproducible quantitation supported by stable isotope-labelled IS, and strong agreement with conventional LC–MS/MS in two independent clinical cohorts. While modest recovery and small sample sizes represent current constraints, the platform achieved clinically meaningful performance within a total analysis time of approximately four minutes. These proof-of-concept findings underscore the potential of Mini-MS to transform TDM of clozapine by enabling rapid, on-demand quantitation. Future work will involve large-scale, multi-centre validation to establish clinical utility and refine the assay workflow for routine implementation. Efforts will focus on improving automation, streamlining data processing, and integrating electronic reporting systems to support adoption in psychiatric care.

Beyond clozapine, this platform could be expanded to include other commonly prescribed antipsychotics including olanzapine, risperidone, paliperidone, aripiprazole, and haloperidol. Furthermore, its application could extend to non-psychiatric drugs with narrow therapeutic windows, where timely and reliable surveillance is equally critical, offering broad potential to transform clinical practice.

## Figures and Tables

**Table 1:** Inter-assay CV, intra-assay CV, and accuracy of clozapine and norclozapine quantification in plasma extract, blood extract, and dried blood spots, using the Mini-MS system analyses with paper capillary spray technology.

Concentration (ngmL^−1^)	Inter CV %	Intra CV %	Accuracy %	Inter CV %	Intra CV %	Accuracy %
	Clozapine in Plasma Extract			Norclozapine in Plasma Extract		
100 (L)	11.18%	13.20%	101%	4.55%	10.40%	94%
500 (M)	12.95%	2.90%	108%	7.10%	4.80%	94%
1000 (H)	8.48%	4.60%	106%	1.60%	3.60%	101%
	Clozapine in Blood Extract			Norclozapine in Blood Extract		
100 (L)	14.05%	16.54%	108%	14.62%	9.15%	114%
500 (M)	11.05%	5.20%	104%	4.41%	5.10%	104%
1000 (H)	7.90%	5.20%	100%	12.87%	2.78%	102%
	Clozapine in DBSs			Norclozapine in DBSs		
167 (L)	9.60%	22.30%	96%	17.80%	13.90%	107%
417 (M)	8.30%	14.20%	108%	18.70%	10.90%	85%
833 (H)	2.90%	21.10%	104%	3.70%	10.90%	116%
1250 (H2)	3.70%	17.60%	101%	4.50%	2.50%	117%

## Data Availability

The original contributions presented in this study are included in the article/Supplementary Materials. Further inquiries can be directed to the corresponding authors.

## Supplementary Materials

The following supporting information can be downloaded at: https://www.mdpi.com/article/10.3390/ph18101549/s1. Figure S1. Workflow of plasma extract, blood extract, and dried blood spot assays, highlighting the estimated hands-on time of each step and assay; Figure S2. MS/MS spectra of analytes at limits of detection. Norclozapine in (A) plasma extract at 0.5 ng/mL, (C) blood extract at 0.5 ng/mL, (E) dried blood spot at 2.5 ng/mL, and clozapine in (B) plasma extract at 0.5 ng/mL, (D) blood extract at 0.5 ng/mL, (F) dried blood spot at 2.5 ng/mL; Table S1: Clinical clozapine and norclozapine concentrations measured by the Mini-MS and conventional LC-MS/MS at Tsinghua University; Table S2: Clinical clozapine and norclozapine concentrations measured by the Mini-MS and conventional LC-MS/MS at New South Wales Health Pathology.

## Abbreviations

The following abbreviations are used in this manuscript:

ADR	Adverse Drug Reaction
DAPI	Discontinuous Atmospheric Pressure Interface
DBS	Dried Blood Spot
IS	Internal Standard
LC-MS/MS	Liquid Chromatography Tandem Mass Spectrometry
LOD	Limit of Detection
LOQ	Limit of Quantification
Mini-MS	Miniature Mass Spectrometry/Spectrometer
nESI	Nano-Electrospray Ionisation
NSWHP	New South Wales Health Pathology
PCS	Paper Capillary Spray
POC	Point-of-Care
TDM	Therapeutic Drug Monitoring
TRS	Treatment-Resistant Schizophrenia
