# Supplementary material for: Rapid On-Demand Point-of-Care Monitoring of Clozapine and Its Metabolite Norclozapine Using Miniature Mass Spectrometry
Source: Pharmaceuticals (Basel). 2025 Oct 14;18(10):1549. doi: 10.3390/ph18101549 (PMC12567311; doi:10.3390/ph18101549)
Supplement: Supplementary file 1 [file pharmaceuticals-18-01549-s001.zip › pharmaceuticals-3884068-supplementary.pdf]

Supplemental materials for

Rapid on-demand point-of-care monitoring of clozapine and its metabolite norclozapine using miniature mass spectrometry

Xiaosuo Wang [1,*], Wei Yi Lew [1], Yang Yang [2], Nan Zhang [3], Jiexun Bu [3], Zhentao Li [4], Michael Fitzpatrick [5], Paul Bonnitcha [5,6], David Sullivan [5,6], Wenpeng Zhang [7], Yu Zheng [8] and John F. O'Sullivan [1,9,*]

1 Cardiometabolic-Medicine Laboratory, Charles Perkins Centre, The University of Sydney, Sydney, NSW, Australia; School of Medical Sciences, Faculty of Medicine and Health, The University of Sydney, Sydney, 2006, Australia; xiaosuo.wang@sydney.edu.au; weiyi.lew@sydney.edu.au; john.osullivan@sydney.edu.au
2 Key Laboratory of TCM Clinical Pharmacy, Shenzhen Bao'an Authentic TCM Therapy Hospital, Shenzhen, Guangdong, 518100, China; yangyanghb@outlook.com
3 PURSPEC Technology (Beijing) Ltd., Beijing, 100084, China; nan.zhang@purspec.cn; jiexun.bu@purspec.cn
4 Department of Pharmacy, The First Affiliated Hospital, Nanchang University, Nanchang, Jiangxi, 330006, China; ndyfy09785@ncu.edu.cn
5 Chemical Pathology, NSW Health Pathology, Royal Prince Alfred Hospital, Camperdown, NSW, 2050, Australia; michael.fitzpatrick1@health.nsw.gov.au; paul.bonnitcha@health.nsw.gov.au; david.sullivan@sydney.edu.au
6 NHMRC Clinical Trials Centre, Sydney Medical School, The University of Sydney, Sydney, NSW, 2006, Australia; david.sullivan@sydney.edu.au
7 State Key Laboratory of Precision Measurement Technology and Instruments, Department of Precision Instrument, Tsinghua University, Beijing, 100084, China; zhangwp@tsinghua.edu.cn
8 Mental Health Service, Croydon Health Centre, Sydney Local Health District, Croydon, NSW, 2132, Australia; yu.zheng@health.nsw.gov.au
9 Department of Cardiology, Royal Prince Alfred Hospital, Sydney, NSW, 2050, Australia; john.osullivan@sydney.edu.au
* Correspondence: XSW, xiaosuo.wang@sydney.edu.au; JOS, john.osullivan@sydney.edu.au

This file includes

- **Materials and Methods**

- **Figure S1:** Workflow of plasma extract, blood extract, and dried blood spot assays, showinging the estimated hands-on time of each step and assay.

- **Figure S2:** MS/MS spectra of analytes at limits of detection. Norclozapine in (A) plasma extract at 0.5 ng/mL, (C) blood extract at 0.5 ng/mL, (E) dried blood spot at 2.5 ng/mL, and clozapine in (B) plasma extract at 0.5 ng/mL, (D) blood extract at 0.5 ng/mL, (F) dried blood spot at 2.5 ng/mL.

- **Table S1:** Clozapine and norclozapine values measured by the Mini-MS system and the conventional LC-MS/MS at Tsinghua University, Beijing

- **Table S2:** Clozapine and norclozapine values measured by the Mini-MS system at the University of Sydney and the conventional LC-MS/MS at NSWHP laboratory, Sydney

Blood and plasma collection

Human blood and plasma samples were collected from healthy CMM laboratory staff, with approvals from the Human Research Ethics Committee in Sydney Local Health District.

Patient blood samples (N=25, non-identifiable) were obtained at the First Affiliated Hospital of Nanchang University. The procedures were reviewed and approved by the Ethics Review Board of Tsinghua University, and informed consent forms were obtained from all participants. All samples were stored and analysed at Tsinghua University.

Patient serum samples (N=30, non-identifiable) were obtained from the NSW Health Pathology (NSWHP) Chemical Pathology Laboratory with ethics approved by the NSWHP Research Governance Office.

Preparation of internal standards, calibration curves and quality control samples

Clozapine-D4 (IS) was dissolved in methanol to form a working concentration of 10 μg/mL and used in sample preparation to form a concentration of 200 ng/mL across all samples. Clozapine and norclozapine standards were first prepared in methanol at working concentrations of 0.25, 1, 4, and 20 μg/mL used to spike into different biological matrices.

For calibration curves in plasma extracts and whole blood extracts, clozapine and norclozapine standards were spiked into samples at varying concentrations (10-1000 ng/mL) with quality control (QC) samples prepared at low (50 ng/mL), medium (500 ng/mL) and high (1000 ng/mL) concentrations of clozapine and norclozapine.

For dried blood spots (DBS), the calibration curves were prepared by adding clozapine and norclozapine standards at various concentrations from 41.7−1250 μg/mL, with QC samples at low (166.7 ng/mL), medium (416.7 ng/mL) and high (1250 ng/mL) concentrations.

Preparations for plasma, whole blood and dried blood spots

For plasma samples, the IS (10 μg/mL, 2 μL) was spiked into plasma or serum (20 μL), followed by addition of clozapine and norclozapine at different concentrations (10–1000 ng/mL). The analyte-spiked plasma samples were extracted with acetonitrile to make the final volume of 100 μL for protein precipitation. After vortexing, the mixture was centrifuged using a palm micro centrifuge for 10 seconds. The resulting supernatant (10 μL) was loaded onto the PCS cartridge, eluted with extraction buffer (100 μL), and submitted for analysis on the Mini MS.

For whole blood samples, fresh blood (5 μL) was spiked with clozapine and norclozapine at varying concentrations (10–1000 ng/mL) and made up to 105 μL with extraction buffer (10 mM ammonium formate and 0.1 % formic acid in acetonitrile) containing IS. With brief vortexing, the mixture was centrifuged using a palm micro centrifuge for 10 seconds. The supernatant (100 μL) was loaded into the PCS cartridge for the Mini MS analysis.

For DBS samples, blood (5 μL) spiked with varying concentrations (41.7–1250 ng/mL) of clozapine and norclozapine was spotted onto a PCS cartridge and dried in an oven (37 °C, 1 min). The DBS was analysed by adding 100 μL of extraction buffer containing IS into the PCS cartridge for Mini MS analysis.

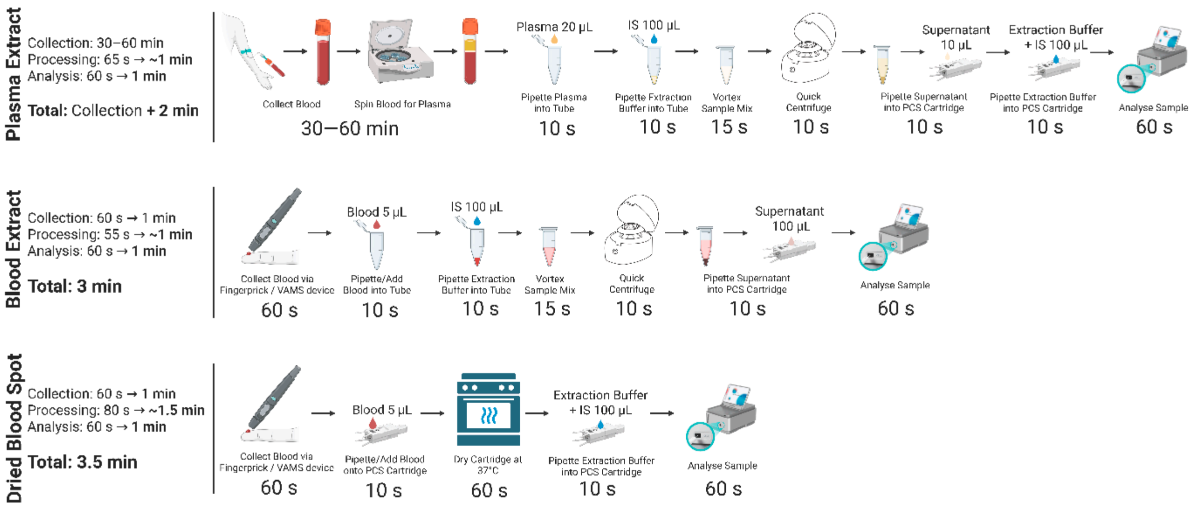

Figure S1. Workflow of plasma extract, blood extract, and dried blood spot assays, highlighting the estimated hands-on time of each step and assay.

Cell Mini Mass Spectrometry and workflow

The Mini-MS system (Cell, PURSPEC Technologies, Beijing, China) consists of a cartridge inlet, DAPI, linear ion trap and detector [30]. Its capabilities include precursor and product ion scans in both positive and negative ionisation modes at a scan range of 50–2000 m/z [31]. Precursor and product ions for clozapine, norclozapine and clozapine-D4 were determined by loading 10 µL of 1 µM chemical standard solutions into a nESI cartridge for Mini-MS analysis. Plasma extracts (10 µL) were loaded into the PCS cartridges in the sample window followed with 100 µL of elution solvent (extraction buffer) into the solvent window for analysis. For the whole blood extracts, extract (100 µL) was directly loaded into the cartridge in the solvent window for analysis. The DBS was treated with 100 µL of elution solvent after drying and subjected for analysis. Spray voltages were applied between 3.8–4.5 kV for the ionization in the positive mode. Collision energies (CE) were optimised by testing a range from 2–8 in "cid_amp". For each analyte, MS1_ISO, MS2 scan and MS2_ISO were performed for precursor and product ions monitoring and quantifications. Specifically, clozapine was detected at m/z 327->270, norclozapine at m/z 313->270 and 313-> 253, and clozapine-D4 at m/z 331->272.

Liquid chromatography tandem mass spectrometry (LC-MS/MS)

A triple quadrupole 7500 mass spectrometer (AB Sciex, Foster City, CA, USA) coupled with a Nexera LC-30AD UHPLC (Shimadzu Corporation, Kyoto, Japan) system was used to detect clozapine and norclozapine, with clozapine–D4 serving as the IS to evaluate the performance of the Mini-MS. Chromatographic separations were achieved on an HPH C18 column (1.8 x 50 mm, 2.7 µm, Agilent Technologies, Santa Clara, CA, USA) with a gradient program. Mobile phase A contained 95% water, 5% acetonitrile, 10 mM ammonium acetate while mobile phase B consisted of 100 % acetonitrile. The gradient was started at 2% B for 0.5 min, increased to 95% at 8 min, and maintained for 1.5 min before decreased to 2% at 10 min. Equilibration at 2% B was carried out for 2 minutes before the next injection. The flow rate was set at 0.2 mL/min. The triple quadrupole MS was operated in positive electrospray ionization mode with the following parameters: spray voltage 3000 V, source temperature at 100 ˚C, curtain gas at 40 psi, ion source gas 1 and 2 were set at 35 and 45 psi, respectively. CEs were optimised using MRM optimisation function in Sciex OS. Clozapine and clozapine-D4 were monitored at m/z326.8->270 and m/z 326.8->192; m/z 331->272 and m/z 192, respectively. Norclozapine shared the same fragment ions as clozapine at m/z 313->270 and m/z 313->192.

Performance evaluation of mini–Mass Spectrometry

The specificity for detecting both precursor and product ions of each analyte using the Mini-MS was validated against the Sciex 7500 LC-MS/MS. Linearity for plasma, whole blood extracts and DBS samples was evaluated across a range of concentrations using the previously described preparation methods. Peak intensity ratios between analytes and IS were plotted against analyte concentrations for linear regression. The limit of quantification was determined as the lowest calibrator concentration with a coefficient of variation (CV) of ≤ 20%, and accuracy between 80-120%. Limit of detection was defined as S/N ≥ 3. Inter- and intra- assay reproducibility were assessed by analysing plasma, whole blood extracts and DBS spiked with low, medium and high analytes concentrations tested in triplicates over at least four different days.

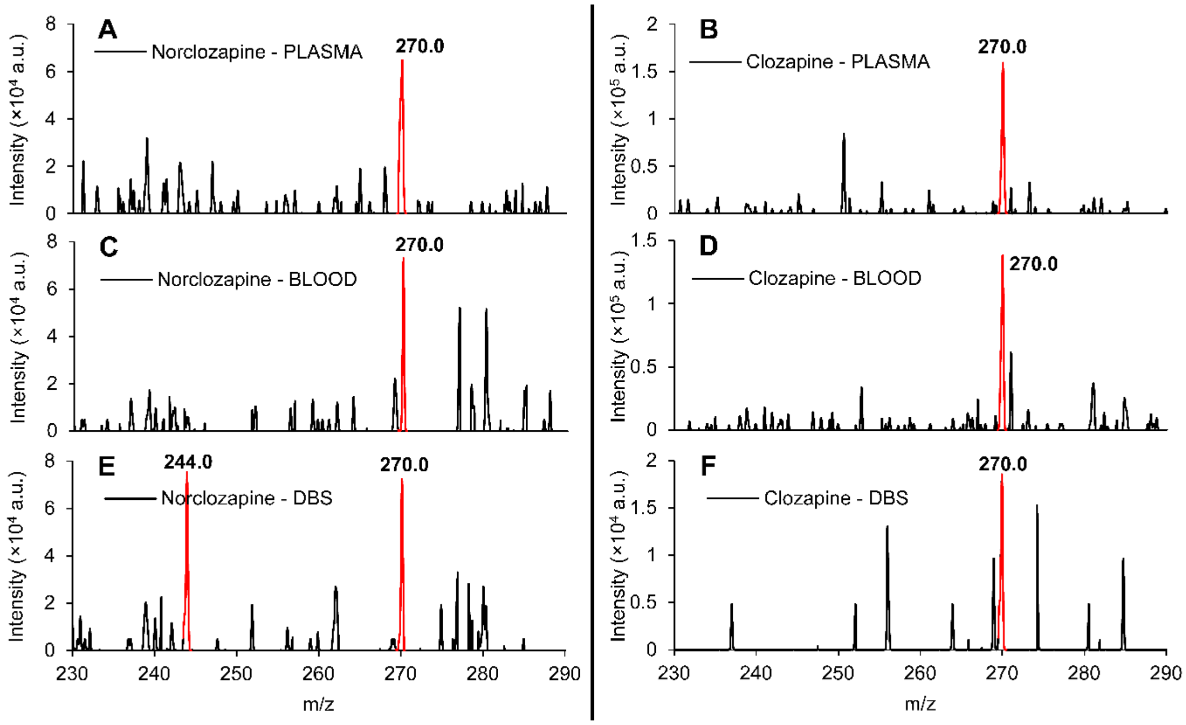

Figure S2. MS/MS spectra of analytes at limits of detection. Norclozapine in (A) plasma extract at 0.5 ng/mL, (C) blood extract at 0.5 ng/mL, (E) dried blood spot at 2.5 ng/mL, and clozapine in (B) plasma extract at 0.5 ng/mL, (D) blood extract at 0.5 ng/mL, (F) dried blood spot at 2.5 ng/mL.

For extraction efficiency, one-step protein precipitation was tested by comparing plasma spiked with low, medium and high concentrations of analytes to samples prepared without plasma. The extraction yield was calculated by comparing the response of the analytes in solvents to the response of the analytes spiked into plasma. The precision tests were calculated using mean of minimal triplicates with standard deviations. The accuracy tests were assessed as:

$$\text{Accuracy \%} = \frac{\text{measured concentration}}{\text{nominal concentration}} \times 100$$

Clozapine-treated patient samples from two hospital sites

Blood samples from patients (N=25) on clozapine therapy were analysed. In brief, blood (100 µL) was extracted with acetonitrile (125 ng/mL IS, 400 µL). After brief vortexing, the mixture (100 µL) was loaded into the PCS cartridge for Mini-MS analysis. In parallel, the mixture of blood sample and acetonitrile containing IS was centrifuged at 12,000 rpm at 4 °C for 5 min. The resulting supernatant (200 µL) was filtered through a 0.22 µm (PTFE) membrane filter before the LC-MS/MS analysis (Shimadzu LCMS8040, Shimadzu Scientific Instruments Inc., Kyoto Japan).

For patient serum samples recruited in Sydney, in brief, each serum sample (20 μL) were extracted with 80 μL of acetonitrile containing 200 ng/mL of IS, as previously described in the plasma preparation section. After centrifugation, the resulting supernatant (10 μL) was loaded into the PCS cartridge, followed by the addition of 100 μL of elution solvents for the Mini-MS analysis. In the NSWHP Chemical Pathology Laboratory, 50 μL of sample was diluted with 50 μL of internal standard solution, 50 μL of 0.01 M Formate buffer and 350 μL of 0.1% formic acid in acetonitrile. Samples were vortex mixed and passed through a phospholipid removal column before centrifugation. A final dilution of 150 μL of supernatant was made with 600 μL of 0.01M formate buffer before transferring to the Shimadzu LCMS-8050 (Shimadzu Scientific Instruments Inc., Kyoto, Japan) for quantitative analysis using positive ion MRM mode.

Table S1. Clozapine and norclozapine values measured by the Mini-MS system and the conventional LC-MS/MS at Tsinghua University.

Sample No.	Clozapine							Norclozapine						
	LC-MS/MS (ng mL^{-1})	Mini-MS ($ngmL^{-1}$)						LC-MS/MS (ng mL^{-1})	Mini-MS ($ngmL^{-1}$)					
		Repeat 1	Repeat 2	Repeat 3	Average	SD	Inter CV %		Repeat 1	Repeat 2	Repeat 3	Average	SD	Inter CV %
1	460.12	453.92	454.69	431.82	446.81	12.99	3%	94.17	92.53	103.66	85.21	93.8	9.29	10%
2	667.06	615.27	616.44	615.45	615.72	0.63	0%	183.81	177.6	186.83	185.28	183.24	4.94	3%
3	140.93	143.91	141.49	137.61	141.00	3.18	2%	24.15	27.53	27.61	25.94	27.02	0.94	3%
4	67.68	70.40	68.04	65.88	68.11	2.26	3%	—	—	—	—	—	—	—
5	334.72	332.79	335.85	337.92	335.52	2.58	1%	40.34	37.45	36.76	41.42	38.54	2.52	7%
6	464.12	483.57	481.88	502.91	489.45	11.68	2%	262.62	232.84	232.69	243.94	236.49	6.45	3%
7	333.56	347.43	377.14	401.49	375.35	27.07	7%	86.54	86.38	79.07	78.77	81.41	4.31	5%
8	—	—	—	—	—	—	—	—	—	—	—	—	—	—
9	25.43	28.60	24.60	27.05	26.75	2.02	8%	—	—	—	—	—	—	—
10	214.52	235.34	235.61	232.87	234.61	1.51	1%	36.21	33.83	29.49	33.51	32.28	2.42	7%
11	446.58	486.89	485.09	472.90	481.63	7.61	2%	35.65	36.95	36.41	38.66	37.34	1.17	3%
12	264.64	281.63	288.98	289.12	286.58	4.28	1%	87.97	86.89	87.19	83.44	85.84	2.08	2%
13	376.34	417.30	412.14	415.25	414.90	2.60	1%	102.23	107.42	107.69	90.49	101.87	9.85	10%
14	527.85	529.63	525.00	521.62	525.41	4.02	1%	63.66	73.55	70.48	72.59	72.21	1.57	2%
15	287.40	269.34	275.03	294.03	279.47	12.93	5%	63.57	69.86	70.78	69.56	70.07	0.64	1%
16	888.01	910.28	897.14	889.42	898.95	10.55	1%	138.1	115.13	128.66	117.54	120.44	7.22	6%
17	141.06	145.91	142.61	147.93	145.48	2.69	2%	36.5	32.45	30.57	32.54	31.85	1.11	3%
18	393.01	377.25	381.56	390.53	383.12	6.77	2%	64.29	58.9	72.44	63.07	64.8	6.93	11%
19	233.21	214.72	214.51	221.08	216.77	3.73	2%	48.62	48.42	46.2	45.71	46.78	1.44	3%
20	103.27	110.78	107.31	104.90	107.66	2.96	3%	—	—	—	—	—	—	—
21	1421.39	1500.65	1481.95	1617.45	1533.35	73.43	5%	297.88	267.09	291.5	291.45	283.35	14.08	5%
22	1610.51	1645.97	1599.89	1664.19	1636.69	33.14	2%	253.25	239.92	278.67	271.34	263.31	20.59	8%
23	192.05	197.64	198.74	198.65	198.34	0.61	0%	47.41	52.18	45.48	50.78	49.48	3.53	7%

24	206.19	215.27	227.32	234.99	225.86	9.94	4%	46.42	43.43	47.23	36.93	42.53	5.21	12%
25	481.40	476.24	490.65	484.88	483.92	7.25	1%	188.39	189.14	190.46	184.33	187.98	3.23	2%

— : Analyte not detected

Table S2: Clozapine and norclozapine values measured by the Mini-MS system at the University of Sydney and the conventional LC-MS/MS at NSWHP laboratory, Sydney

Sample No.	Clozapine		Norclozapine	
	LC-MS/MS (ngmL^{-1})	Mini-MS (ngmL^{-1})	LC-MS/MS (ngmL^{-1})	Mini-MS (ngmL^{-1})
1	374	394.4	105	<100
2	184	214.6	86	<100
3	342	430.6	175	149.2
4	594	602.6	284	316.4
5	279	319.2	162	161.3
6	686	933.9	311	439.4
7	766	848.7	461	584.4
8	171	206.8	127	104.9
9	348	243.7	197	105.4
10	117	140.7	127	97.4
11	211	239.7	56	<100
12	240	253.7	169	130.7
13	665	790.9	381	351.2
14	209	224.3	69	<100
15	961	1034.1	882	1168.1
16	246	240.8	230	178.2
17	256	245.2	93	<100
18	241	224	183	147.1
19	560	598.9	277	297.7
20	386	449.2	213	239.8
21	861	827.3	337	363.3
22	618	701.6	386	464.1
23	634	536.7	284	347.7
24	1174	1255.2	580	706.2
25	411	345.8	344	227.6
26	182	218.8	153	125.2
27	246	288.2	200	136.1
28	866	824.2	502	471.2
29	500	477	168	159
30	46	67.1	<28	<100
